# COVID-19 pandemic and thyroid diseases

**DOI:** 10.3389/fendo.2026.1784314

**Published:** 2026-04-22

**Authors:** Majd Irsheid, Nitzan Burrack, Merav Fraenkel, Eli Hershkovitz, Uri Yoel

**Affiliations:** 1 Faculty of Health Sciences, Ben-Gurion University of the Negev, Beer-Sheva, Israel; 2 Soroka University Medical Center, Beer-Sheva, Israel; 3 Clinical Research Center, Soroka University Medical Center, Beer-Sheva, Israel; 4 Endocrinology Unit, Soroka University Medical Center, Beer Sheva, Israel; 5 Pediatrics Department & Pediatric Endocrinology Unit, Soroka University Medical Center, Beer-Sheva, Israel

**Keywords:** COVID-19, Graves’ disease, Hashimoto’s thyroiditis, SARS-CoV-2, subacute thyroiditis, thyroid diseases

## Abstract

**Background:**

The COVID-19 pandemic has been associated with various autoimmune manifestations. Several studies have suggested a potential association between COVID-19 and thyroid diseases (TDs); however, findings remain inconclusive and are primarily based on relatively small studies. Population-level data examining the differential impact of the pandemic on specific thyroid conditions are scarce.

**Objective:**

To examine the incidence patterns of Hashimoto’s Thyroiditis (HT), Graves’ Disease (GD), and Subacute Thyroiditis (SAT) during the COVID-19 pandemic compared to the pre-pandemic period.

**Methods:**

We conducted a population-based retrospective cohort study using interrupted time series analysis of adults (≥16 years) in the Clalit Health Services southern district of Israel from January 2018 to December 2022. New cases of TDs were identified using either ICD-9 codes, laboratory results, medication dispensing data or a combination of them. Monthly disease-specific incidence rates were compared between pre-pandemic (January 2018-February 2020) and pandemic (March 2020-December 2022) periods, with adjustment for seasonal variations.

**Results:**

Among 4,765 incident TD cases identified, 3,731 (78.3%) had HT, 698 (14.6%) had GD, and 336 (7.1%) had SAT. The mean age was similar across groups (43–45 years) with consistent female predominance (77%). Interrupted time series analysis revealed a significant 30% increase in HT incidence during the pandemic period (IRR 1.30, 95% CI 1.04-1.64, p=0.023), which began prior to the national vaccination campaign. GD showed a non-significant upward trend suggestive of a possible increased incidence (IRR 1.66, 95% CI 0.99-2.79, p=0.054). Conversely, SAT demonstrated a significant 54% reduction in incidence (IRR 0.46, 95% CI 0.21-0.99, p=0.049).

**Conclusions:**

The COVID-19 pandemic was associated with a significant increase in HT incidence and an unexpected decrease in SAT. These findings highlight the heterogeneous impact of the pandemic on different TDs.

## 1 Introduction

Since its initial outbreak in December 2019, Coronavirus Disease 2019 (COVID-19), caused by Severe Acute Respiratory Syndrome Coronavirus 2 (SARS-CoV-2), has emerged as a global pandemic, infecting over 778 million individuals and resulting in more than 7 million deaths worldwide. In Israel, there were more than 4.8 million confirmed cases, and 12,707 recorded deaths to date (1). The primary system affected by COVID-19 is the respiratory system, manifesting clinical symptoms ranging from asymptomatic or mild flu-like conditions to severe cases such as Acute Respiratory Distress Syndrome (ARDS). However, accumulating evidence underscores COVID-19 as a systemic infection capable of affecting multiple organ systems. Moreover, emerging evidence also indicates that COVID-19 infection might trigger autoimmune responses in genetically predisposed individuals, with multiple reports of autoimmune-like phenomena affecting various organ systems, and showing greater risk for autoimmune disease including thyroid disease following COVID-19 infection (2–7).

Thyroid diseases (TDs), encompassing autoimmune and inflammatory disorders such as Hashimoto’s thyroiditis (HT), Graves’ disease (GD), and subacute thyroiditis (SAT), have shown rising incidence rates in recent years (8). HT and GD are autoimmune TDs characterized by distinct immunological and clinical profiles. HT involves chronic lymphocytic infiltration and autoantibody production against thyroid antigens, leading primarily to hypothyroidism (8, 9), whereas GD results from thyroid-stimulating hormone receptor antibodies (TRAbs) causing hyperthyroidism (10). SAT typically presents as a painful inflammation following viral infections with a classical triple phase response of thyroid function tests starting with thyrotoxicosis, followed by hypothyroidism and ultimately returning to euthyroidism in most cases. The etiology of SAT remains incompletely understood but involves both viral triggers and genetic susceptibility (11).

Recent literature increasingly highlights potential associations between COVID-19 and various thyroid conditions, suggesting that infection may precipitate autoimmune and inflammatory TDs, including HT, GD, and SAT (4, 5, 7, 12–15). Studies indicate thyroid gland involvement post-infection, manifesting as thyrotoxicosis, SAT, or altered thyroid hormone profiles (16, 17). A meta-analysis suggested a prevalence of thyroid dysfunction in up to 15% of COVID-19 patients, with higher rates among those with severe disease (18). SAT has emerged as a potential manifestation, typically occurring within weeks following infection and characterized by neck pain, fever, and transient thyrotoxicosis (19–22). A prospective cohort study found that thyroid peroxidase (TPO) antibody positivity was twice as prevalent among COVID-19 survivors compared to controls, with ultrasonographic findings consistent with thyroiditis in most antibody-positive patients. Recent large-scale electronic health record studies from the United States and International networks have shown higher incidence of both hyperthyroidism and hypothyroidism after documented SARS-CoV-2 (5, 23–25). Despite the growing body of evidence linking COVID-19 to thyroid dysfunction, significant knowledge gaps remain regarding the temporal dynamics and specific patterns of TD incidence during the pandemic. Most existing studies are limited by small sample sizes, cross-sectional designs, or focus on hospitalized patients, potentially missing the broader population-level impact of SARS-CoV-2 on thyroid health. Moreover, most population-based studies relied solely on diagnostic codes without further validation with lab/imaging criteria, potentially conflating between destructive thyroiditis, GD, and chronic Hashimoto’s, and may capture prevalent but previously undiagnosed disease.

The present study seeks to expand our understanding of the interplay between COVID-19 and thyroid dysfunction by investigating the incidence patterns of HT, GD, and SAT, comparing the pre-pandemic period (January 2018 to February 2020) to the pandemic period (March 2020 to December 2022). By examining a large-scale, population-based cohort, we aim to generate evidence-based insights that will clarify the scope of SARS-CoV-2–related thyroid involvement.

## 2 Materials and methods

### 2.1 Study design and population

We conducted a population-based retrospective cohort study using an interrupted time series design to evaluate the association between the COVID-19 pandemic outbreak in March 2020 and the incidence of three distinct TDs: HT, GD, and SAT. The study population comprised all adult members (aged ≥16 years) of Clalit Health Services (CHS) in the southern district of Israel between January 1, 2018, and December 31, 2022. CHS is Israel’s largest health maintenance organization, providing healthcare services to approximately 67% of the 750,000 residents in the Negev region, ensuring comprehensive population coverage.

### 2.2 Data sources

Electronic health records were extracted from the CHS database using the MDClone platform (www.mdclone.com), a secure data-sharing platform that enables access to de-identified clinical data. Patients’ data was extracted based on diagnostic codes documented according to the International Classification of Diseases, 9th revision (ICD-9).

### 2.3 Case definitions

We applied a specific pre-defined method to identify new cases of each of the three TDs. For all three conditions, only the first occurrence of disease after January 1, 2018, was considered an incident case.

#### 2.3.1 HT

Given that HT represents the predominant cause of hypothyroidism and is frequently coded as hypothyroidism in clinical practice, we employed an inclusive definition of all new hypothyroidism cases. Incident cases were identified using ICD-9 codes 245.2x (HT), 244.x (acquired hypothyroidism), and an internal CHS chronic diagnosis code for “hypothyroidism” derived from an algorithm integrating medical summaries from patient’s files, laboratory test results, and medication purchase data. Patients were excluded if they had: (1) a history of thyroidectomy before the study period, (2) any hypothyroidism diagnosis before 2018, (3) positive thyroid antibodies (anti-TPO or anti-thyroglobulin) before 2018, or (4) levothyroxine prescriptions before 2018.

#### 2.3.2 GD

Cases were identified through either: (1) a specific diagnosis of GD (ICD-9: 242.0x; toxic diffuse goiter/GD), or (2) a diagnosis of thyrotoxicosis [ICD-9: 242 (thyrotoxicosis with or without goiter), excluding 242.3x (Toxic nodular goiter unspecified type) and 242.0x (toxic diffuse goiter/GD)] accompanied by dispensing of antithyroid medications (methimazole or propylthiouracil) within one month before or after diagnosis. Previous diagnoses of GD recorded before 2018 resulted in exclusion.

#### 2.3.3 SAT

Cases were identified using: (1) a specific diagnosis of SAT (ICD-9: 245.1), or (2) thyrotoxicosis diagnosis (excluding codes 242.3x and 242.0x) combined with either non-steroidal anti-inflammatory drug (NSAID) dispensing within two weeks of diagnosis or prednisone dispensing within two weeks after diagnosis. Patients who received antithyroid medications (methimazole, propylthiouracil) within three months of diagnosis were excluded to avoid misclassification with other thyroid conditions.

### 2.4 Ethical considerations

The study protocol was approved by the Institutional Review Board of Soroka University Medical Center (SOR 0294-21) and was conducted in accordance with the Declaration of Helsinki. Given the retrospective nature of the study using de-identified data, informed consent was waived.

### 2.5 Statistical analysis

Descriptive statistics were employed to characterize the study population. Categorical variables were presented as frequencies and percentages, while continuous variables were presented as mean ± standard deviation (SD). Baseline demographic and clinical characteristics of the three TD groups (HT, GD, and SAT) were compared to identify differences in patient profiles. Additionally, we examined temporal trends in the sociodemographic characteristics of the study population across the five-year period (2018-2022).

To assess the association of the COVID-19 pandemic on TDs incidence, we employed an interrupted time series (ITS) analysis. Monthly incidence rates were calculated per 100,000 population. The ITS analysis was designed to examine seasonal patterns and model incidence rate ratios (IRRs). This estimation was based on Poisson regression modelling of monthly incident count of HT, GD, and SAT events. The models incorporated harmonic terms (sine and cosine functions) representing 12-month and 6-month cycles to adjust for seasonality of annual and semi-annual patterns. To evaluate the specific impact of the pandemic, the ITS models were parameterized with a period indicator to estimate the immediate level change at the onset of the COVID-19 pandemic (March 2020), and an interaction term between period and time to account for changes in the slope (trend) thereafter. The primary outcome measure, reported as Incidence Rate Ratios (IRRs) with 95% confidence intervals, corresponds to the period indicator and reflects the immediate level change associated with the pandemic period. The regression model included an offset defined by the population size at the beginning of each year. Finally, model adequacy was confirmed by assessing overdispersion (via dispersion tests) and evaluating residual autocorrelation (via the Durbin-Watson test), neither of which showed significant violations.

Monthly incidence patterns were visualized using locally weighted scatterplot smoothing (LOWESS) curves. All statistical tests were two-sided, and we considered results to be statistically significant when the P value was less than 0.05. All statistical analyzes were conducted using R v.4.1.2 (R Foundation for Statistical Computing, Vienna, Austria, http://www.R-project.org).

## 3 Results

During the five-year study period (2018-2022), we identified 4,765 new cases of TDs among adults aged ≥16 years in the CHS southern district of Israel. Of these, 3,731 (78.3%) were diagnosed with HT, 698 (14.6%) with GD, and 336 (7.1%) with SAT. The overall crude incidence rates over the 5-year study period were 175.3, 32.8, and 15.8 cases per 100,000 person-years for HT, GD, and SAT, respectively.

Baseline demographic characteristics of the three groups are presented in **Table 1**. The mean age at diagnosis was similar across groups (43–45 years), with a consistent female predominance (77%). Population-level temporal trends demonstrated a stable demographic composition across the study period (**Table 2**). The mean age remained stable around 43 years old throughout the five years. Similarly, ethnic distribution showed minimal variation, with Arab representation stable at 26-27% and Jewish representation at 70-72% across all years. The sex distribution of the general population showed remarkable consistency, with females comprising 52% of the population in each year of the study.

**Table 1:** Sociodemographic and clinical characteristics of the three study groups: HT, GD, and SAT.

Variable		HT (N = 3,731)	GD (N = 698)	SAT (N = 336)
Age at Diagnosis	Years	43.0 ± 20.2	45.2 ± 17.6	45.1 ± 18.0
Ethnicity	Arab	913(24%)	190(27%)	65(19%)
	Jewish	2,704(72%)	487(70%)	261(78%)
	Other	114(3%)	21(3%)	10(3%)
Sex	Female	2,875(77%)	534(77%)	260(77%)
	Male	856(23%)	164(23%)	76(23%)
COVID-19 Vaccine Doses Before Index Diagnosis	1 Dose	1,299(35%)	205(29%)	121(36%)
	≥2 Doses	1,172(31%)	187(27%)	111(33%)
Prior infection	N(%)	475(13%)	78(11%)	44(13%)
Diagnosis Year	2018	454(12%)	124(18%)	70(21%)
	2019	585(16%)	132(19%)	55(16%)
	2020	741(20%)	162(23%)	60(18%)
	2021	902(24%)	143(20%)	70(21%)
	2022	1,049(28%)	137(20%)	81(24%)

Number (%); Mean ± SD.

HT, Hashimoto’s Thyroiditis; GD, Graves’ Disease; SAT, Subacute Thyroiditis.

**Table 2:** Demographic characteristics of Clalit Health Services patients included in study cohort throughout study period.

Variable		2018 (N = 393,453)	2019 (N = 409,506)	2020 (N = 425,544)	2021 (N = 441,878)	2022 (N = 458,419)
Age		43.2 ± 18.9	42.9 ± 19.0	42.6 ± 19.0	42.5 ± 19.0	42.4 ± 19.0
Ethnicity	Arab	101,658 (26%)	107,311 (26%)	113,095 (27%)	118,238 (27%)	123,232 (27%)
	Jewish	281,848 (72%)	291,598 (71%)	301,075 (71%)	311,269 (70%)	321,498 (70%)
	Other	9,947 (3%)	10,597 (3%)	11,374 (3%)	12,371 (3%)	13,689 (3%)
Sex	Female	205,713 (52%)	213,492 (52%)	221,317 (52%)	229,362 (52%)	237,042 (52%)
	Male	187,740 (48%)	196,014 (48%)	204,227 (48%)	212,516 (48%)	221,017 (48%)

Number (%); Mean ± SD.

**Figure 1:**
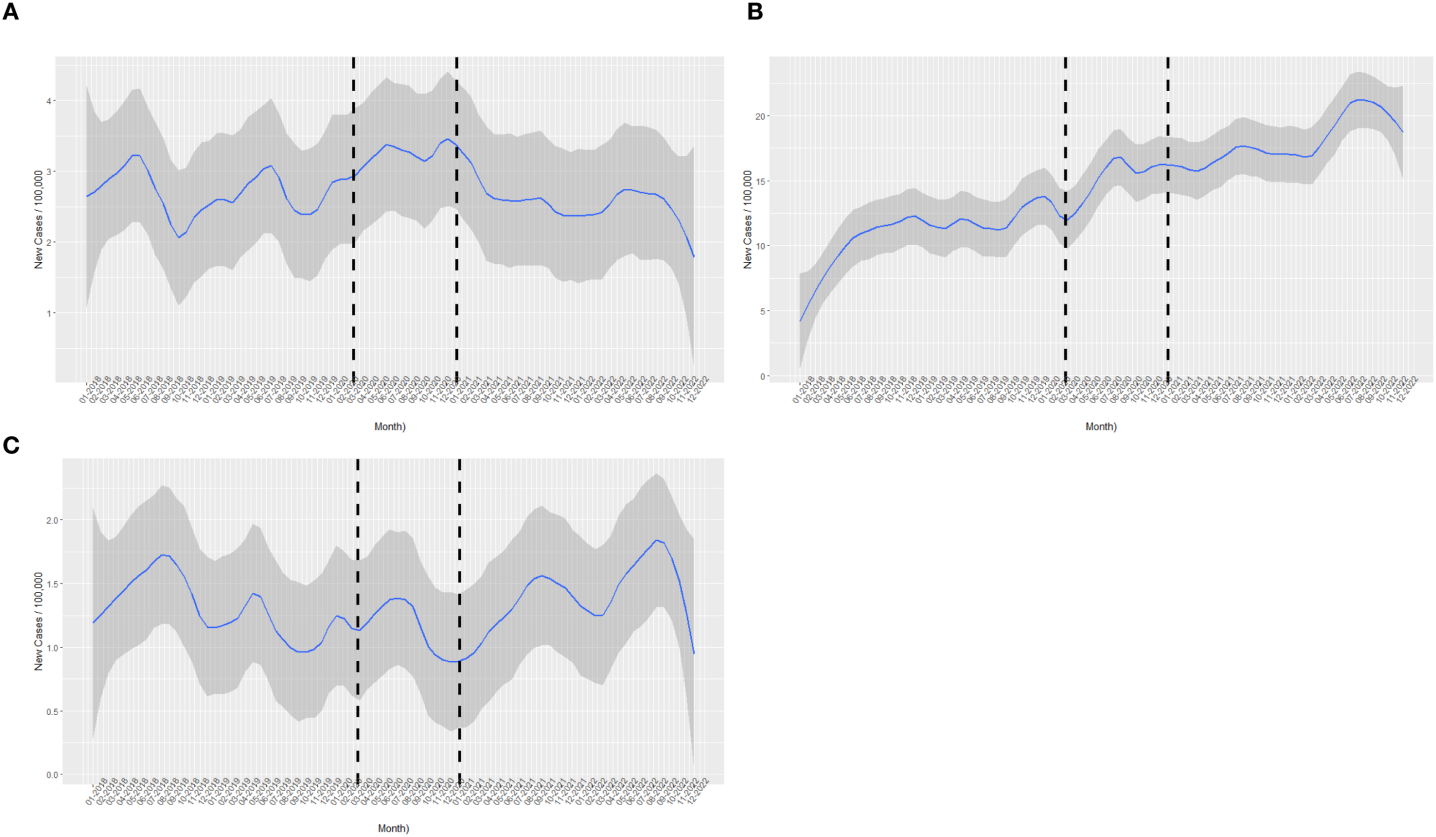
Locally Weighted Scatterplot Smoothing (LOWESS) of monthly disease-specific incidence per 100,000 CHS members; vertical dashed lines mark March 2020 (pandemic onset) and December 2020 (vaccine rollout). The figures show the incidence fluctuations timeline for **(A)** Graves’ Disease, **(B)** Hashimoto’s Thyroiditis, and **(C)** Subacute Thyroiditis. The blue line represents the Locally Weighted Scatterplot Smoothing fitting curve of the regression describing the monthly incidence. LOWESS curves are provided for descriptive visualization only and were not used for inferential statistical modelling.

The interrupted time series analysis revealed distinct and divergent patterns of TD incidence in relation to the COVID-19 pandemic onset (**Table 3**, **Figures 1A–C**). GD showed a non-significant upward trend suggestive of a possible increase (IRR 1.66, 95% CI 0.99–2.79, p=0.054) (**Figure 1A**). Also, there was a statistically significant 30% increase in HT incidence compared to the pre-pandemic baseline (IRR 1.30, 95% CI 1.04-1.64, p = 0.023). In absolute terms, HT incidence increased from 115.4 cases per 100,000 population in 2018 to 228.8 cases per 100,000 in 2022. This increase in HT diagnoses began prior to the commencement of the national vaccination program in December 2020, as illustrated in **Figure 1B**. Interestingly, SAT demonstrated a 54% reduction in incidence during the pandemic period (IRR 0.46, 95% CI 0.21-0.99, p = 0.049), with **Figure 1C** showing a marked decline of SAT prior to the commencement of the national vaccination program in December 2020, followed by an increase several months after vaccination initiation. Absolute SAT incidence remained relatively stable over the study period (17.8 per 100,000 in 2018 vs. 17.7 per 100,000 in 2022), while GD incidence showed minimal change (31.5 vs. 29.9 per 100,000, respectively).

**Table 3:** Interrupted time series analysis and incidence rate ratios for HT, GD, and SAT.

Variable	IRR	95% CI	p-value
HT	1.30	1.04-1.64	**0.023**
GD	1.66	0.99-2.79	0.054
SAT	0.46	0.21-0.99	**0.049**

Boldface type indicates p<0.05.

Reference Group: pre-pandemic (Jan 2018–Feb 2020).

IRR, Incidence Rate Ratio; CI, Confidence Interval.

HT, Hashimoto’s Thyroiditis; GD, Grave’s Disease; SAT, Subacute Thyroiditis.

The temporal distribution of TD diagnoses revealed dynamic changes over the five-year study period (**Table 1**). For HT, there was a progressive increase in case numbers from 454 (12%) in 2018 to 1,049 (28%) in 2022, with the most substantial increases occurring in 2021 (24%) and 2022 (28%). This temporal pattern aligns with the pandemic-associated increase identified in the ITS analysis. GD and SAT remained relatively stable throughout study period, though showing some year-to-year variation.

## 4 Discussion

In this population-based study, we investigated the association between the COVID-19 pandemic and the incidence of autoimmune and inflammatory TDs. Our analysis of 4,765 new TD cases over five years revealed distinct patterns: a significant 30% increase in HT incidence during the pandemic period, a non-significant 66% increase in GD, and an unexpected 54% reduction in SAT cases. Importantly, this ITS analysis evaluates population-level temporal associations and does not assess individual-level temporal relationships between confirmed SARS-CoV-2 infection and TD onset. Moreover, direct causal inference regarding SARS-CoV-2 infection cannot be established from this design of our study.

The observed increase in HT and the non-significant upward trend in GD incidence align with several studies, including population-based studies demonstrating increased incidence of new-onset autoimmune diseases after SARS-CoV-2 infection — including HT and GD — with risk gradients that scale with acute COVID-19 severity, suggesting that SARS-CoV-2 infection may trigger autoimmune responses in genetically predisposed individuals, leading to an increased incidence of TD (2–5, 7, 14, 15, 23–25). Our study suggests an increased incidence of HT and indicates a possible increase in GD during the pandemic period, did not investigate the correlation between covid-19 severity and its impact on thyroid disorders. Importantly, prospective COVID cohorts have also demonstrated increased prevalence of TPO and thyroglobulin antibodies, thyroiditis-compatible ultrasound patterns, and subtle structural changes in the gland several months after infection — even when overt thyroid dysfunction was absent — supporting the possibility of subclinical thyroid autoimmunity triggered or amplified by SARS-CoV-2 (15, 26). Notably, the surge in HT diagnoses preceded the national vaccination campaign, suggesting a primary association with the pandemic itself rather than vaccination-related effects. This temporal relationship is particularly important given concerns about vaccine-induced autoimmunity (7, 19, 27, 28), and supports the hypothesis that SARS-CoV-2 infection may trigger autoimmune responses in genetically predisposed individuals through molecular mimicry, cytokine-mediated thyroid injury, and direct viral effects on ACE2-expressing thyroid tissue (27, 29, 30). However, in our retrospective study, the effect of COVID-19 vaccination on the thyroid could not be determined and differentiated from COVID-19 infection itself.

The unexpected reduction in SAT incidence during the pandemic period presents a paradox. While SAT typically follows viral infections (11), our data showed a 54% decrease during the pandemic. These findings reflect the heterogeneity described in prior clinical reports, with some showing no increase in SAT while others identified increase in SAT (both painful and painless variants) within three months of infection as well as among long-COVID-19 patients (31–34). Several hypotheses may explain this finding. First, public health measures including masking, social distancing, and lockdowns likely reduced transmission of other common respiratory viruses that traditionally trigger SAT. Second, the predominance of SARS-CoV-2 may have competitively inhibited other viral infections (35, 36). Third, healthcare-seeking behaviors during the pandemic may have led to underdiagnosis of milder SAT cases, as patients avoided medical facilities due to infection concerns (37, 38). Fourth, it has been shown that post-COVID-19 SAT may be difficult to identify due to potential absence of classic symptoms (e.g. neck tenderness), as well as cross‐over of common clinical features between COVID‐19 and thyrotoxicosis (21). The subsequent increase in SAT cases following vaccination initiation, as shown in our temporal analysis, aligns with isolated reports of vaccine-associated SAT (19, 30), though the absolute numbers remain low. In addition to biological explanations, healthcare-system–related factors may have contributed to the observed reduction in SAT incidence. Reduced access to outpatient care during lockdown periods may have delayed or prevented evaluation of patients with neck pain. Furthermore, overlap between COVID-related thyrotoxicosis and classical SAT presentations may have introduced diagnostic ambiguity, potentially influencing case classification.

Our study’s strengths include its population-based design, comprehensive case ascertainment through multiple diagnostic criteria, and rigorous ITS analysis accounting for seasonal variations. By using diagnostic coding together with medication-dispensing data to classify HT, GD, and SAT, this study addresses a major limitation of previous large-scale electronic health record studies, which largely relied on diagnostic codes alone. The five-year study period provided adequate baseline data for robust comparisons. The stable demographic composition of the study population throughout the observation period strengthens confidence in the observed associations. Nevertheless, several limitations warrant consideration. First, the potential for ascertainment bias cannot be excluded, as healthcare utilization patterns changed dramatically during the pandemic. Initial lockdowns and fear of infection may have delayed diagnosis, while heightened awareness following media reports about thyroid effects of COVID-19 and vaccines may have increased diagnostic vigilance. Second, our inclusive definition of HT, which incorporated all incident hypothyroidism cases based on administrative diagnostic codes, may have captured some non-autoimmune etiologies. Although this approach reflects real-world clinical coding practices and increases sensitivity, it may have led to misclassification. Such misclassification could potentially overestimate the incidence of autoimmune thyroiditis, particularly during periods of increased diagnostic testing. This limitation should be considered when interpreting the observed increase in HT incidence. We mitigated this potential bias through multi-criteria case definitions incorporating laboratory results and medication dispensing. Third, Increased thyroid function testing during the pandemic, whether due to post-infection laboratory screening or heightened awareness of endocrine complications, may have contributed to increased detection of subclinical or mild hypothyroidism. This diagnostic intensity could have led to partial overestimation of HT incidence. Conversely, reduced healthcare access during lockdown periods and avoidance of medical facilities may have contributed to underdiagnosis of milder SAT cases, particularly those not requiring urgent care. Forth, despite its large sample size, the study may have been underpowered to detect the smaller effect size of GD which was a less common condition compared to HT. Finally, given the ecological nature of the design, direct causal inference regarding SARS-CoV-2 infection and TD onset cannot be established.

In conclusion, this study suggests a heterogeneous association between the COVID-19 pandemic and TD incidence, with significant increases in HT, a non-significant upward trend in GD, and reductions in SAT. These findings emphasize the complex relationship between pandemic conditions and autoimmune and inflammatory TDs, highlighting the need for continued vigilance in monitoring and managing thyroid dysfunction in the post-pandemic era. Our results contribute to the growing body of evidence characterizing the multisystem effects of COVID-19 and inform clinical practice regarding screening and management of TDs in pandemic affected individuals.

## Data Availability

The dataset belongs to Clalit HMO. Requests to access these datasets should be directed to elih@bgu.ac.il.
